# Semaglutide Reduces Odorant-Driven Chemotaxis and Reproductive Output in *Caenorhabditis elegans*

**DOI:** 10.17912/micropub.biology.002168

**Published:** 2026-09-23

**Authors:** Sophia Kousnetzov, Angela C. Incollingo Rodriguez, Richard B. Lopez, Jagan Srinivasan

**Affiliations:** 1 Biology and Biotechnology and Psychological Sciences , Worcester Polytechnic Institute, Worcester, MA, United States; 2 Psychological & Cognitive Sciences Program, Department of Social Science & Policy Studies and MS Program in Neuroscience, Worcester Polytechnic Institute, Worcester, MA, United States; 3 Biology and Biotechnology, Worcester Polytechnic Institute, Worcester, MA, United States

## Abstract

Glucagon-like peptide-1 receptor agonists (GLP-1RAs) reduce food intake and body weight in humans, partly via neural circuits governing reward and energy homeostasis. Using *Caenorhabditis elegans* as a tractable model, we examined neurobehavioral and physiological effects of semaglutide, a long-acting GLP-1RA. Wild-type N2 animals exposed to semaglutide (1 μM and 10 μM) showed significantly reduced chemotaxis toward the volatile odorant diacetyl. Semaglutide also reduced egg-laying and delayed larval development at both concentrations tested. These phenotypes demonstrate that semaglutide alters multiple *C. elegans* behaviors and developmental processes, supporting its utility as a model system for investigating GLP-1RA mechanisms at cellular resolution.

**Figure 1. Semaglutide reduces food-cue chemotaxis and reproductive output in C. elegans .** **(A)** Chemotaxis index (CI) of wild-type N2 worms toward diacetyl (0.5%) following exposure to vehicle ( OP50 + 5% acetic acid), 1 μM semaglutide, or 10 μM semaglutide. Both 1 μM and 10 μM semaglutide significantly suppressed chemotaxis relative to vehicle-treated controls. No clear dose-dependent gradient was observed between the two active concentrations. **(B)** Egg-laying (fecundity) across vehicle, 1 μM, and 10 μM semaglutide treatment groups. Egg-laying decreased at both semaglutide concentrations. **(C)** Egg hatch rate, measured as the fraction of eggs that hatched following exposure to vehicle, 1 μM semaglutide, or 10 μM semaglutide. Semaglutide treatment significantly increased egg hatching at both concentrations relative to vehicle-treated controls **(D)** Developmental progression measured as the number of F1 progeny reaching L4 stage, counted 48 hours after egg synchronization on experimental plates. Wild-type N2 animals on vehicle control plates showed normal developmental timing, while semaglutide-treated animals showed a significant reduction in L4 progeny at both concentrations tested, indicating delayed developmental progression. In panel A, each data point represents 60-80 worms, and in panels B–D, each data point represents the progeny or eggs derived from a single worm. Bars show mean ± SEM. Statistical comparisons were performed relative to vehicle-only controls using one-way ANOVA with Dunnett's multiple-comparisons post-test in GraphPad Prism. *P < 0.05, and **P < 0.01

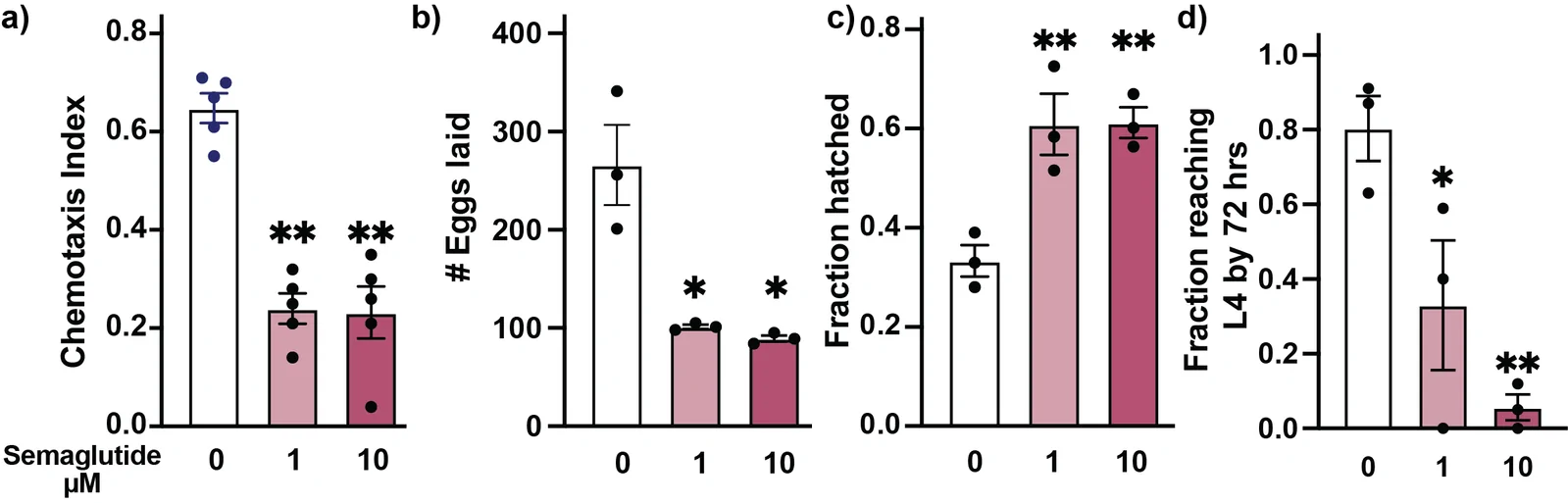



## Description

Glucagon-like Peptide-1 receptor agonists (GLP-1RAs) have emerged as transformative pharmacological agents, initially developed for the management of type 2 diabetes and now widely prescribed for obesity treatment. Approximately 12% of American adults (~39 million individuals) currently use a GLP-1RA (Tomiyama, 2025). Beyond their well-established metabolic benefits—improved glycemic control, sustained weight reduction, and favorable cardiometabolic outcomes—GLP-1RAs increasingly demonstrate neurobehavioral effects that extend beyond simple energy balance (Tomiyama, 2025). GLP-1 receptors are expressed throughout the central nervous system in regions governing appetite, satiety, and reward, including the hypothalamus, the nucleus tractus solitarius in the brainstem, the ventral tegmental area, and the nucleus accumbens (Baggio & Drucker, 2007; Farr et al., 2016).

Emerging clinical and preclinical evidence indicates that GLP-1RAs attenuate cue reactivity—the coordinated neural and behavioral response to food-related stimuli—and reduce maladaptive eating behaviors (Klausen et al., 2022). Semaglutide in particular has been reported to reduce self-described "food noise," a colloquial term for persistent, cue-driven food-related rumination, and to diminish the motivational salience of high-caloric food cues in human neuroimaging studies (Blundell et al., 2017). Despite this progress, the specific circuit-level and molecular mechanisms mediating these neurobehavioral actions remain poorly understood, partly because of the complexity of mammalian reward systems and the limited resolution of current neuroimaging approaches.

*Caenorhabditis elegans* offers a complementary experimental platform for dissecting the neurobiology of feeding behavior and nutrient sensing. Its 302-neuron connectome is fully mapped, its metabolic and nutrient-sensing pathways are highly conserved with mammals, and its behavioral repertoire includes robust and quantifiable chemotaxis assays (Bargmann et al., 1993; Brenner, 1974; Meneely et al., 2019; Sengupta & Samuel, 2009). Odor-driven chemotaxis toward attractive odorants such as diacetyl — produced by bacterial food sources — provides a tractable proxy for food-cue reactivity. Although GLP-1RAs have not previously been examined in this system, conserved insulin/IGF-like signaling ( DAF-2 / DAF-16 ), AMPK, and TOR pathways in *C. elegans* coordinate feeding behavior, reproduction, and developmental fate with nutritional state (Kenyon, 2010). We therefore asked whether exogenous semaglutide exposure alters appetitive chemotaxis and reproductive physiology in *C. elegans* , and if so, whether the resulting phenotypes parallel those predicted by GLP-1RA action in mammals.

To assess the effect of semaglutide on food-cue approach behavior, we performed a standard four-quadrant diacetyl chemotaxis assay on synchronized N2 young adults exposed to OP50-seeded NGM plates supplemented with vehicle (5% acetic acid), 1 μM semaglutide, or 10 μM semaglutide. A one-way ANOVA revealed a significant effect of semaglutide concentration on the chemotaxis index (CI) toward diacetyl ( *p* = .018; Figure 1A ). Marked suppression of chemotaxis was observed at both 1 μM and 10 μM relative to vehicle-treated OP50-fed controls, with no clear dose-dependent gradient between these two concentrations. These data indicate that semaglutide reduces chemotaxis toward attractive food-associated odorants, a behavioral shift analogous to the GLP-1-mediated attenuation of food-cue salience observed in mammals.

Semaglutide exposure also affected reproductive output and developmental progression (Figures 1B-D). Semaglutide treatment reduced egg-laying, although the effects observed at the two concentrations were not significantly different ( Figure 1B ). In contrast, egg hatch rate was significantly increased at both semaglutide concentrations ( Figure 1C ). Despite increased hatching, developmental progression was delayed, with fewer F1 progeny reaching the L4 stage by 72 h in semaglutide-treated groups, the most pronounced delay at 10 μM ( Figure 1D ). Collectively, these findings indicate that semaglutide exposure alters multiple physiological processes in *C. elegans* , including sensory behavior, reproductive output, embryonic hatching, and postembryonic developmental timing.

The reduction in diacetyl chemotaxis observed following semaglutide exposure in *C. elegans* is consistent with Glucagon-like Peptide-1 receptor agonist-mediated suppression of reward-related and approach circuitry observed in mammalian models and human neuroimaging studies. Even at low micromolar concentrations, semaglutide significantly reduced diacetyl chemotaxis, demonstrating that semaglutide alters olfactory behavior in *C. elegans* at concentrations relevant to its pharmacological activity. The absence of a concentration-response gradient between 1 μM and 10 μM may reflect a ceiling effect at these concentrations or indicate that the observed effects are not mediated by a specific receptor-dependent mechanism. Future dose-ranging experiments across a broader concentration range will be necessary to distinguish these possibilities. The unexpectedly low hatch rate in vehicle-treated controls relative to semaglutide-treated groups likely reflects a fecundity-hatch rate trade-off rather than a direct pro-hatching effect of semaglutide. High-fecundity vehicle plates accumulate a greater density of eggs, which in *C. elegans* is associated with local food depletion and crowding-dependent signaling (e.g., ascaroside pheromones) that can suppress hatching efficiency independent of embryo quality.

Several important caveats must be considered. First, *C. elegans* lacks a canonical GLP-1 receptor ortholog, and the specific molecular target of semaglutide in this organism remains unidentified. While the peptide may be cross-reacting with endogenous neuropeptide receptors, the observed phenotypes could also stem from alterations in the OP50 bacterial food source, indirect modulation of conserved metabolic sensors, or microbiome-mediated effects. Second, because these initial behavioral assays utilized micromolar concentrations to overcome the nematode cuticle barrier, we cannot definitively rule out that the phenotypic plateau reflects non-specific physiological stress or toxicity. Furthermore, it remains to be determined whether the reduced chemotaxis reflects a specific attenuation of food-cue salience or a secondary behavioral deficit linked to the observed developmental delays. Future studies utilizing nanomolar dose-response curves, coupled with genetic loss-of-function screening of candidate neuropeptide receptors, will be required to definitively distinguish specific pharmacological target engagement from generalized stress. Nonetheless, these preliminary results support the utility of *C. elegans* as a tractable *in vivo* model for investigating the neurobehavioral impacts of GLP-1 receptor agonists.

## Methods

**Strains and maintenance**

Wild-type N2 *C. elegans* were maintained on 6-cm NGM plates seeded with *E. coli* OP50 at 20°C and passaged every four days according to standard protocols (Brenner, 1974). Synchronized populations were generated by hypochlorite treatment and allowed to develop to the L4/young adult stage before transfer to experimental plates.

**Semaglutide exposure**

Worms were transferred to NGM plates seeded with OP50 supplemented with semaglutide at final concentrations of 1 μM or 10 μM, or to vehicle control plates ( OP50 supplemented with 5% acetic acid, the semaglutide solvent). Worms were maintained on these plates for 24 hours prior to behavioral and reproductive assays.

**Chemotaxis assay**

Odorant chemotaxis was assessed using a four-quadrant assay adapted from Bargmann et al. (1993). Worms (60–80 per plate) were washed three times in M9 buffer and placed at the center of chemotaxis agar plates. Diacetyl (0.5% in ethanol) and water were applied to opposing quadrants (5 μL each), with 0.5 M sodium azide (3 μL per quadrant) added as an anesthetic to immobilize worms upon arrival. Plates were incubated at 20°C for 60 min, after which worms in the attractant, control, and origin zones were counted. The chemotaxis index (CI) was calculated as:

CI = (N _attractant_ − N _control_ ) / N _total_

Assays were performed in five independent biological replicates. Statistical significance was assessed by one-way ANOVA.

**Fecundity and developmental staging**

For fecundity assays, individual L4/young adult hermaphrodites were transferred to experimental plates and egg counts were recorded daily. Age-synchronized L4 hermaphrodites were individually transferred to experimental plates containing either the semaglutide treatment or a precisely matched vehicle control (diluted 5% acetic acid stock). All assays were conducted at 20°C. To accurately assess fecundity and completely prevent the confounding mixing of generations, each mother was transferred to a fresh experimental plate every 24 hours until egg-laying ceased. For fecundity measurements, the total number of eggs laid per 24-hour period was recorded immediately after the mother's transfer. For developmental staging, the freshly laid eggs on these plates were then incubated at 20°C for an additional 48 to 72 hours. Following this incubation period, the number of F1 progeny that successfully reached the L4 stage was counted.

## Reagents

**Table:** 

*C. elegans N2*	Wild-type Bristol strain	Caenorhabditis Genetics Center (CGC)
Semaglutide	GLP-1 receptor agonist; MW 4113.6 Da; dissolved in 5% acetic acid	Adipogen A01847 5mg
Diacetyl (2,3-butanedione)	Olfactory attractant; 0.5% v/v in ethanol	Sigma-Aldrich
* E. coli OP50*	Standard *C. elegans* food source; uracil auxotroph	CGC
